# Complexity Science in Domestic Abuse Literature: A Systematic Scoping Review

**DOI:** 10.1177/15248380251316225

**Published:** 2025-02-24

**Authors:** Sarah Blake, James Nobles

**Affiliations:** 1 The University of Bristol, UK; 2 Leeds Beckett University, UK

**Keywords:** domestic violence and abuse, intimate partner violence, complexity science, systems science, complexity theory, scoping review, interdisciplinary research, complex adaptive systems, complex and wicked problems, holistic approaches

## Abstract

Complexity science is an interdisciplinary paradigm that helps people understand how outcomes, such as domestic violence and abuse (DVA), arise from within complex adaptive systems. This study aims to identify how complexity science has been applied in DVA literature. A systematic scoping review was conducted, searching across academic databases and Google for articles. Articles published from 1990 to 2020, written in English, had DVA partner abuse as a focus, and used complexity science as a focus or theoretical background to the paper, were considered for inclusion. Data was extracted and narratively synthesized in an iterative manner. Twenty-one studies were included, originating predominantly from the United States and New Zealand, and published mainly after 2009. Approximately 70% of authorships were comprised of interdisciplinary teams. Most papers strongly incorporated complexity science as a methodological approach and applied methods, such as systems modeling (agent-based modeling or systems dynamics modeling), aligning with computer science or engineering disciplines. Some used complexity theory combined with qualitative techniques (interviews or discourse analysis) strongly associated with social sciences research. Methods and findings were heterogeneous and often explored interactions between parts of the system and the subsequent phenomena that emerged from these interactions. Complexity science can: (a) support a holistic understanding of DVA; (b) combine different perspectives; (c) encourage interdisciplinary teams to work collaboratively around an issue such as DVA; (d) identify leverage points to assist in targeting scarce resources; (e) help predict emergent phenomena and unexpected consequences of policy change.

## Background

The UK government defines domestic violence and abuse (DVA) as any “incident or pattern of incidents of controlling, coercive, threatening behavior, violence or abuse between those aged 16 or over who are, or have been, intimate partners or family members regardless of gender or sexuality” (Home Office, 2012). Over 26% of women and 15% of men in the United Kingdom will experience domestic abuse in their lifetime (ONS, 2017), with 152 family homicides related to DVA in 2019 (ONS, 2019). DVA disproportionately affects women and children (Hester & Lilley, 2014). However, there are high numbers of male victims, and it is thought that the actual number is likely to be underestimated as many do not report the abuse (Bates, 2020). DVA is estimated to cost the UK economy around £66 billion annually (Oliver et al., 2019).

DVA research often adopts either the feminist perspective, which has been beneficial for understanding inequality via the lens of patriarchy, the dynamics of power and control, and the forms in which controlling behavior can present itself (Bancroft, 2003; Dobash & Dobash, 1992; Stark, 2007), or psychological perspectives. These have been useful for understanding the contribution of behavioral conditioning, attachment theory, trauma, and intergenerational links to abuse (Johnson & Ray, 2016; Murray, 2006). However, despite the relevance of these perspectives to DVA research they only offer a partial outlook.

Abuse could be understood as a person’s mindset that is characterized by entitlement, narcissism, a deluded constructed vision of the world, and short-term gratification at the expense of others (Bancroft, 2003). The abusive mindset, which is often a prerequisite for the manifestation of DVA, can also emerge as other forms of abuse (Carne et al., 2019), such as child sexual abuse (Bancroft, 2007), bullying (Gibbons, 2019; Ruiz-Narezo et al., 2020), oppressive regimes (Sini, 1999), terrorism (Smith, 2019), and mass shootings (Casciani & De Simone, 2021). Understanding that the abusive mindset can emerge in multifaceted forms assists us in drawing attention to its complexity.

DVA is sometimes framed as a “complex” or “wicked” problem (Tracy et al., 2023; Young-Wolff et al., 2016). When issues are presented as “wicked” problems, they can shift and adapt over time (Page, 2009). A wicked problem is complex, multifaceted, and enduring (Carne et al., 2019). There is an ambiguity of causality (Grohs et al., 2018), and the problem can metamorphosize, leading to unexpected outcomes. For instance, if gender equality was achieved, there are no guarantees that DVA would end, as suggested by the conundrum of the Nordic Paradox, whereby Sweden has experienced higher rates of DVA despite its progressive gender equality policies (Gracia & Merlo, 2016; Gracia et al., 2019). To gain a holistic understanding of domestic abuse, which can also incorporate the shifting nature of complex problems, a paradigm such as complexity science may be a useful ideology. This paradigm has gained significant traction in recent years within the public health domain (Rusoja et al., 2018).

The complexity science paradigm proves difficult to define as it encompasses a wide range of terms and frameworks used across multiple disciplines (Castellani & Gerrits, 2024); however, there are core concepts that help to provide some common ground (see Table 1). Complexity science can provide an ontological and epistemological perspective, language, tools, and frameworks that can help break down disciplinary boundaries and see the world in different ways. Many of the traditional approaches using reductionist techniques fall short of what is needed to understand an increasingly global, unpredictable, and interconnected world (Rusoja et al., 2018).

**Table 1.** Complexity Science Terms Used to Describe CASs.

Term	Description
CAS	Collections of interacting, self-organizing, adaptive components (agents).
Nonlinearity	In nonlinearity, the outputs are not in proportion to their inputs. A small change can escalate into something much bigger or something large-scale can make little difference.
Interconnectedness	CASs consist of many parts which interact and are self-autonomous.
Path dependency	Path dependence is when actions are based on what has occurred in the past.
Emergence	Emergent phenomena are the bottom-up creation and patterns that arise spontaneously from interactions within the CAS. They are not easily predicted and can take unexpected manifestations.
Self-organization	A process whereby some form of order emerges out of interactions between components that were initially disordered.
Feedback	An acceleration or dampening down of activity created by one component in the system impacting another component. A chain of cause and effect which can control or regulate the system. (A) can affect (B) but (B) can also affect (A) creating a feedback loop.
Unintended consequences	Unplanned consequences that could be beneficial or harmful.
Stable/unstable systems Tipping point	The system may be stable and in “equilibrium.” Changes that occur may balance out and not disrupt the whole system. Systems can transition from one state of equilibrium to another. The point that this transition accelerates irreversibly is called a “tipping point.”
Leverage points	Points in a system where a small shift in one area can create large changes across the system.

*Source*. Adapted from Meadows (2008), Sturmberg et al. (2014), and Egan et al. (2019).

*Note*. CAS = complex adaptive system.

Complexity science takes a holistic perspective to understand an issue, explore the behavior of interdependent elements (tangible and intangible parts of a system), and describe changes within complex adaptive systems (CASs). CASs have self-organizing dynamic properties that interact with each other, sometimes producing unpredictable and significant events to emerge, influencing the system and outcomes as a whole (Page, 2009; Rusoja et al., 2018; The Health Foundation, 2010). Complexity science can be used to understand complex problems and could prove to be a valuable addition to existing DVA research by enhancing the potential to embrace multiple perspectives and consider the complexity of DVA (Carne et al., 2019).

The abusive mindset tends to follow certain characteristics and patterns. It tends to target vulnerability, and perpetrators often abuse those around them who are vulnerable to them in that context. The role of perpetrator and victim can be flexible. They can switch depending on the context and those present at that given time, and there is a possibility that yesterday’s victim could become tomorrow’s perpetrator (Ruiz-Narezo et al., 2020). Abuse is not static; if it cannot *emerge* in one form, it may move into another. It can *self-organize* into networks, creating the positive *feedback loops* and reinforcement it needs to thrive. Understanding the *interconnectedness* and *nonlinear* patterns of abuse and interventions may give us an insight into problems that may be difficult to obtain if focusing on isolated elements.

DVA also tends to cluster within networks (Tracy et al., 2023). The network of people surrounding a perpetrator, with an abusive mindset, could be seen as a CAS. As well as the person(s) being abused, those in the system of interest may consist of various agents, such as family, friends, pets, colleagues, professionals, bystanders, and institutional and legal bodies. The interactions between these agents may give rise to *emergent* events, such as homicides, job loss, or a new life as someone finds another system to reside in. Some of these events may be predictable, others less so. Furthermore, the systems within which a perpetrator operates (i.e., social circles, employment, and family) are often well-established and rather resilient to change, meaning that a significant degree of intervention is required to fundamentally change how this system works. For example, a custodial sentence, functioning at the perpetrator (i.e., individual level), may offer little impact if not offering community-level group programs addressing the underlying psychopathology of an offender. However, a multifaceted reform, at a societal level, may cause longer-term systemic changes to occur.

Many public health issues are too complex to “solve” by a singular “downstream” action, and international research teams have proposed the need for using complexity science within interdisciplinary teams worldwide (Thompson et al., 2016). This study aims to identify the extent complexity science has been used in DVA literature, with specific interest in the following questions: (a) What are the characteristics of the included studies? and (b) How has the complexity science paradigm been applied in the studies?

The current scoping review therefore builds on four existing reviews (Carey et al., 2015; Rusoja et al., 2018; Sturmberg et al., 2014; Thompson et al., 2016), which have explored complexity science in public health, enabling comparison between the DVA and public health literature. An additional review by Tracy et al. (2023) has studied the use of systems science to understand the risk, outcomes, interventions, and community responses associated with domestic and gender-based violence. Our work also builds on and complements this by expanding search terms (e.g., complexity theory) and including a broader range of article types (e.g., grey literature).

## Methodology

### Study Design

A systematic scoping review was undertaken to develop a comprehensive overview of the literature (Gough et al., 2017). It integrated a broad range of disciplines, perspectives, and methods, providing an overview of an emerging evidence base.

### Search Strategy

The search strategy was informed by the study’s aims and previous reviews (Carey et al., 2015; Rusoja et al., 2018; Sturmberg et al., 2014; Thompson et al., 2016), and was reported on using the PRISMA checklist (PRISMA, 2020). A full list of search terms for academic databases, Google Scholar, and Google are available in Supplemental Material A. Search terms included those related to DVA and complexity science. The following databases were searched in June 2021: Sociological Abstracts, Web of Science Core Collection, IBSS, PsycINFO, PubMed, CINAHL, and Cochrane. The search was limited to texts published between 1990 and 2020 due to preliminary investigations and the work of the four previous reviews.

### Screening

**Figure 1.**
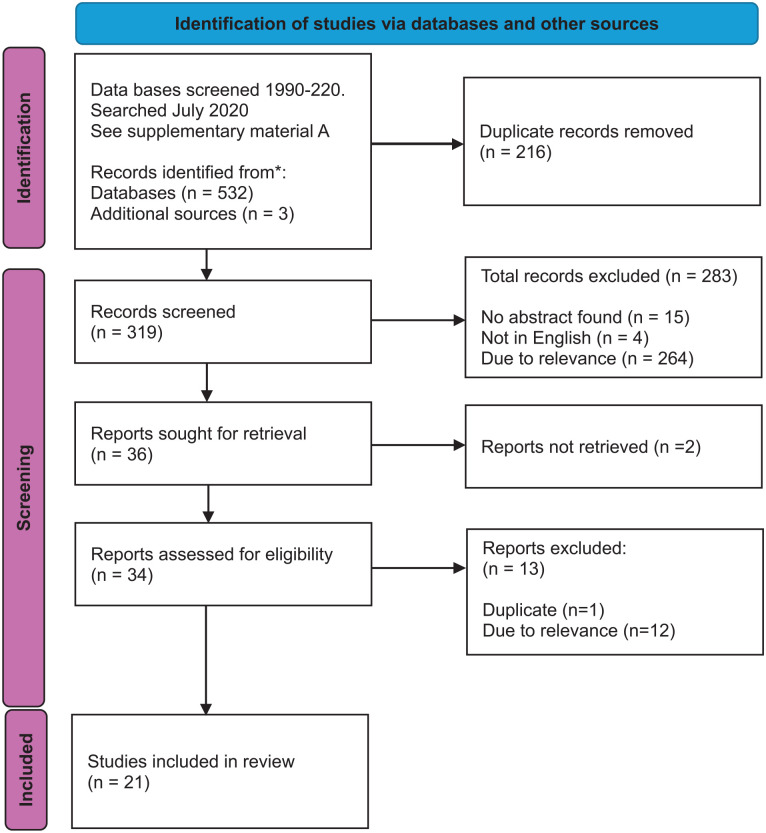
PRISMA flow diagram.

The lead author screened all papers (Figure 1). A total of 532 articles were transported into EndNote. 216 papers were duplicates, leaving 316 for the title and abstract screening against the inclusion and exclusion criteria (Table 2). DVA is a broad subject that includes sexual violence, honor-based violence, and child abuse. Due to its extensive scope, we focused this study on partner abuse, and we excluded the use of other holistic approaches sometimes used to understand DVA, such as the ecological model, family systems theory, whole systems approaches, and social network analysis.

**Table 2.** Inclusion and Exclusion Criteria.

Inclusion Criteria	Exclusion Criteria
- Published from 1990 to 2020 - English language - CS or CAS concepts used as a focus or as a theoretical background to inform the paper - DVA as a focus of the paper - Peer and non-peer-reviewed articles - Conference proceedings - Full texts available	- Articles published before 1990 (limits set in search where possible) - Articles published after 2020 - Non-English language articles - Studies referring to other holistic approaches (ecological model, family systems theory, whole systems approaches, and social network analysis) - Studies which focus on child abuse only - Studies relating to sexual violence and abuse only - Studies where CS are only featured as keywords - PhD/dissertation thesis

*Note*. CAS = complex adaptive system; DVA = domestic violence and abuse.

Fifteen papers were excluded as they had no identifiable abstract, four were excluded as they were not in English, and 264 because they were not the relevant focus of the study (Supplemental Material B). Thirty-three papers were included to go forward to the full-text review stage. In the “Google Scholar” search, all generated references were examined. Five of the six articles were duplicates, leaving one for inclusion in the full-text review.

The grey literature was searched via Google. Due to the large number of documents retrieved, the first 50 documents in each search combination were screened (250 total). Twenty-five were screened against the inclusion and exclusion criteria. Only one was not duplicated and deemed suitable for the final review (see Supplemental Material A). Bibliographies of included studies were also screened. The hand search identified 21 potential additional papers, of which one was retained.

A total of 36 papers were in the full-text review (see Supplemental Material C). Two papers could not be located. After the full-text review and screening, 21 papers (linked to 13 research studies) were included (see Table 3).

**Table 3.** The Lens, Category, and Depth of the Included Studies.

Lens	Author(s)	Date	Title	Origin	Depth
Theoretical lens (*n* = 2)	Olive	2016a	Classificatory multiplicity: Intimate partner violence diagnosis in emergency department consultations	United Kingdom	Limited
	Olive	2016b	First contact: Acute stress reactions and experiences of emergency department consultations following an incident of intimate partner violence	United Kingdom	Limited
Position pieces (*n* = 5)	Gear et al.	2018a	Advancing complexity theory as a qualitative research methodology	New Zealand	Strong
	Gear et al.	2018c	Utilizing complexity theory to explore sustainable responses to intimate partner violence in health care	New Zealand	Strong
	Burge et al.	2014	Safely Examining Complex Dynamics of intimate partner violence	United States	Strong
	Foot et al.	2015	measuring the effectiveness of “whole of system” response to prevent family violence	New Zealand	Strong
	Carne et al.	2019	Using systems thinking to address intimate partner violence and child abuse in New Zealand	New Zealand	Strong
Methodological approach (*n* = 13)	Gear et al.	2018b	Exploring the complex pathway of the primary care response to intimate partner violence in New Zealand	New Zealand	Strong
	Gear et al.	2019	Exploring sustainable primary care responses to intimate partner violence in New Zealand: Qualitative use of complexity theory	New Zealand	Strong
	Burge et al.	2016	Using complexity science to examine three dynamic patterns of intimate partner violence	United States	Strong
	Katurndahl et al.	2019a	Agent-based modeling of day-to-day intimate partner violence	United States	Limited
	Katurndahl et al.	2020	Modeling women’s need for action in violent relationships	United States	Limited
	Burge et al.	2019	The dynamics of partner violence and alcohol use in couples: Research methods	United States	Strong
	Katurndahl et al.	2019b	Psychometrics of the violence nonlinear dynamics scale	United States	Limited
	Guidi et al.	2016	Stochastic agent-based models of intimate partner violence	Italy	Moderate
	Hovmand and Ford	2009a	Sequence and timing of three community interventions to domestic violence	United States	Strong
	Hovmand and Ford	2009b	computer simulation of innovation implementation strategies	United States	Moderate
	Hovmand et al.	2009	Victims arrested for DVA: Unintended consequences of arrest policies	United States	Strong
	Hovmand et al.	2012	Group model- building scripts as a collaborative planning tool	United States	Moderate
	Deutsch et al.	2020	Community-based system dynamics modeling of sensitive public health issues: Maximizing diverse representation of individuals with personal experiences	United States	Strong
Analytical lens (*n* = 1)	Makleff et al.	2020	Applying a complex adaptive systems approach to the evaluation of a school-based intervention for intimate partner violence in Mexico	Mexico	Strong

*Note*. DVA = domestic violence and abuse.

### Data Extraction, Analysis, and Synthesis

The data extraction was designed around the research questions and criteria applied in the four previous public health reviews (Carey et al., 2015; Rusoja et al., 2018; Sturmberg et al., 2014; Thompson et al., 2016). We used a narrative synthesis, given the heterogeneity within this field. Narrative syntheses are able to bring data together from different types of research designs. It is a technique to help the reviewer conduct a synthesis that is systematically grounded in the included studies. Themes were created from “common concepts” that were identified in more than one study within the data extraction table. The themes and codes were developed during analysis through an iterative and inductive approach as the process unfolded, and the reviewer was informed by the data, research questions, and background reading (Gough et al., 2017). A common language was applied across the data to help compare results.

Data extraction focused on the following: Paper *Characteristics* included the title, authors, year of publication, and the country of origin of the study. It also stated where the papers were being cited and indicated the readership and influence of the original papers included in the review. *Framing* included disciplines, epistemological perspectives, ontological approaches, and stakeholders. *The function* encapsulated how complexity science was used within the studies (position pieces, theoretical lens, analytical lens, methodological approaches), adapted from Carey et al. (2015). *Outcomes* included the findings and recommendations of the studies. Data were extracted by the lead author into Microsoft Excel.

We allocated studies to one of three categories to understand the extent to which complexity science was used within each study. (a) *Strong use*: Complexity science informs or is embedded throughout the paper, and there is a clear explanation of what the concepts mean, including an alignment between different aspects of the paper. (b) *Moderate use*: Complexity science concepts are referred to in several sections of the paper, and concepts are used or explained in either a written form, via modeling and diagrams, or both. (c) *Limited use*: The paper mentions complexity science in the abstract or one section, but the concepts are not explained or embedded into the paper.

## Results

**Figure 2.**
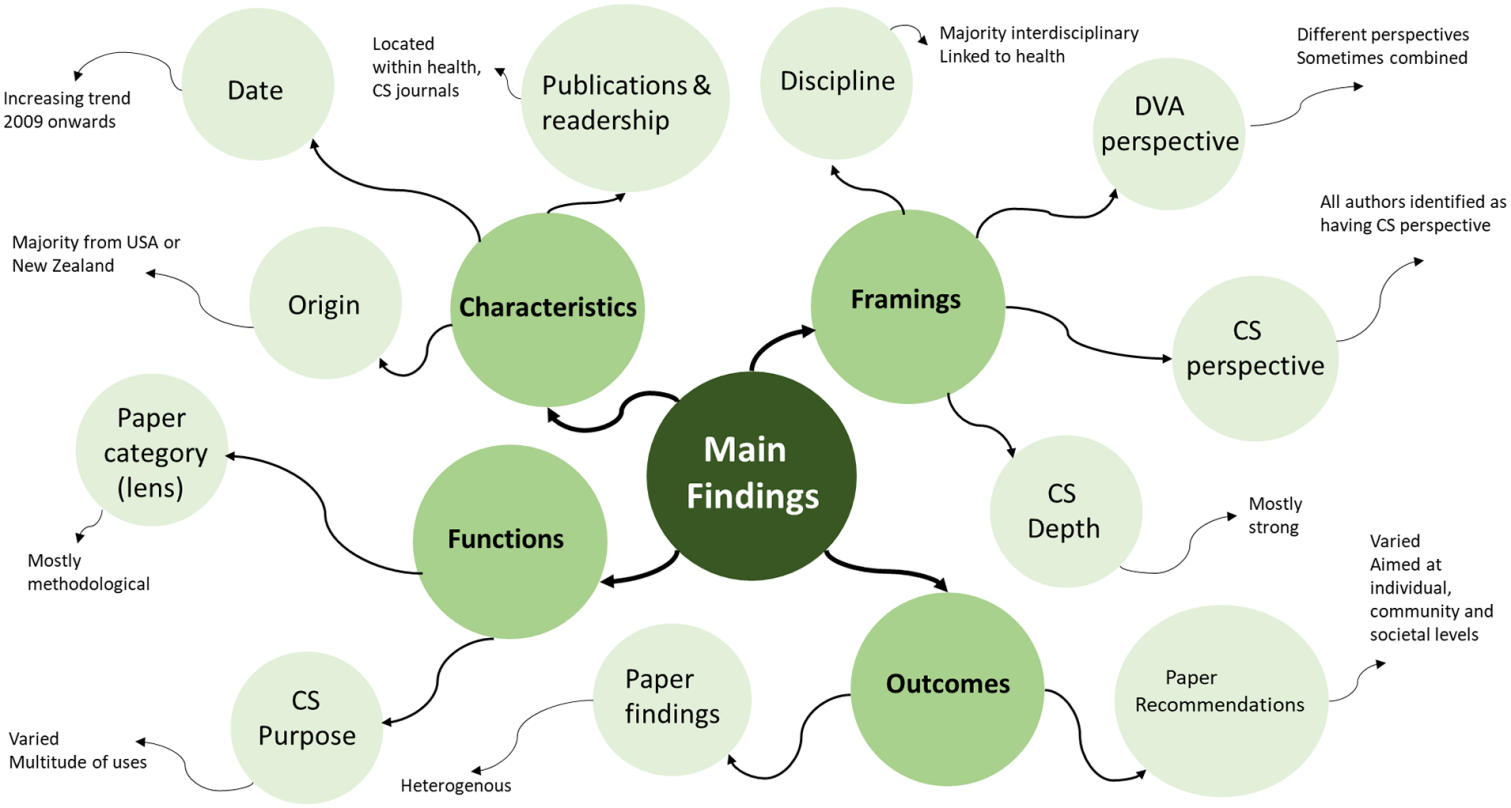
Overview of themes.

An overview of the main findings from this scoping review is available in Figure 2. The findings will be presented thematically based on (a) the characteristics of the included papers, (b) how DVA was framed, (c) how complexity science was applied, and (d) the findings and recommendations.

### Characteristics of Included Papers

Slightly more than half of the papers (52%) originated from the USA and approximately 30% from New Zealand. Papers from the USA were predominantly written by two teams (Katerndahl and Burge et al.; Hovmand et al.). Four papers from New Zealand were produced by one research team (Gear et al.) Two papers came from the United Kingdom, and one from Mexico and Italy, respectively. Most studies (57%) were published between 2018 and 2020. No articles were published between 1990 and 2009 (see Table 3).

There was a wide range of disciplines reflected in the papers. Most authors (73%) were linked to establishments that have been coded (via institutions referenced on the papers) under the umbrella of “health.” This is a diverse category and includes violence and trauma research, family planning, social work, and pediatrics. Leadership disciplines accounted for 6% and engineering for 5% of the authorship’s disciplines, with a smaller number coming from various disciplines, including physics, environmental science, and education. Fifteen of the papers (71%) were authored by interdisciplinary teams. Further information is available in Supplemental Material D.

Articles have been published in a variety of journals and sources: relating to health and complexity science (26%), independent research (13%), management (13%), psychology and behavioral sciences (13%), and DVA or violence (9%). See Supplemental Material E.

As of June 2021, the 21 included studies had been cited 429 times in other work. Of these citations, 116 (27%) were cited within DVA literature. The most frequently cited paper (Hovmand et al., 2012—182 citations) focused on group model building for systems dynamics modeling. However, the papers that were cited were seldom from the DVA literature.

### Framings of Included Papers

The selected papers embedded their framing of complexity science to varying degrees, referred to as the “depth” of complexity science use. Table 3 shows that the majority of the papers had complexity science strongly embedded into the papers (*n* = 13), and a few used it in a moderate (*n* = 3) or limited (*n* = 4) manner (see “Methodology” section). The majority of studies were often very clear, and authors often explained, at length, what complexity science was and how it could be a helpful perspective to understand DVA. DVA perspectives were generally less obvious throughout the papers and often had to be deduced from “clues” in the text relating to language, participants, or information in the background reading.

Studies from New Zealand took an inclusive frame when describing DVA. They refer to DVA as “family violence” or “Intimate Partner Violence.” In the works of Gear et al. (2018a, 2018b, 2018c, 2019), DVA is stated to be a public health issue, and they make a strong case for the use of complexity science consistently throughout the papers. The perspective of post-structuralism was also brought into their narrative. Carne et al. (2019) focused the whole paper on the urgent need for a complexity science perspective to understand DVA. Multiple DVA perspectives are applied within this paper to discuss the interconnections between violence, the co-occurrence of different forms of violence, the life course trajectory of violence, and the social conditioning of gender norms.

USA studies were inconsistent in their framing. Terminology tended to be gender inclusive. Intimate partner violence is the most common term used. Hovmand and Ford (2009a, 2009b) used the terms “victim” and “offender” predominantly in the context of mandatory arrest policy. They discussed the disproportionate impact on female victims in some papers, with no reference to male victims. Burge et al. (2014, 2016, 2019) and Katerndahl et al. (2019a, 2019b, 2020) papers were unclear in their DVA framing. Papers used several theories of DVA associated with the feminist perspective, including the cycle of violence and the Duluth model of power and control. However, they also used family systems theory, which is associated with the psychological model. Several papers discussed the disproportionate effect on women (Katerndahl et al., 2016, 2020); in others, research recognized gender symmetry in abuse (Burge et al., 2019). Some studies have recruited female-only participants (Burge et al., 2014, 2016), while one included male and female couples to understand the dynamics between both parties (Burge et al., 2019).

The UK papers (Olive, 2017a, 2017b) focused on “gender-based violence,” which aligns with the feminist perspective. These papers were linked to complexity science, critical realism, postmodernism, and the sociology of a diagnosis. All study participants were female. The study from Mexico (Makleff et al., 2020) used the “Duluth” model and critiqued the work by Gear et al. for not placing enough emphasis on gender norms. The paper from Italy (Guidi et al., 2016) incorporated elements of feminist and psychological approaches, setting them within the understanding of the complexity science paradigm.

### Functions of Included Papers

Function relates to how complexity science was applied within the included studies. Articles were coded into four “lens” categories based on the earlier work of Carey et al. (2015) to demonstrate their approach to complexity science (Table 3). *Theoretical lenses* (*n* = 2) used complexity science to inform the study’s theory. They may have mentioned complexity science in the abstract or background reading. *Position pieces* (*n* = 5) advocated for the application of complexity science and may describe how this could be achieved. Research that used complexity science as a method was coded within the *methodological approach* category (*n* = 13). Methods included agent-based modeling (Guidi et al., 2016; Katerndahl et al., 2019a, 2020), systems dynamic modeling (Hovmand et al., 2009), group model building (Hovmand et al., 2012), complexity theory combined with qualitative interviews (Gear et al., 2019), or discourse analysis (Gear et al., 2018b). Research that applied complexity science to the analysis section, post hoc, was put into the category of *analytical lens* (*n* = 1).

The purpose of complexity science varied across all papers. Some papers used complexity science to develop new insights (Gear et al., 2018a, 2018b; Hovmand & Ford, 2009b). For example, DVA responsiveness could be reconceptualized as a CAS to understand the interactions between system elements and documents that produce emergent phenomena, such as discourse (Gear et al, 2018b). Others used it to understand the emergence of sustainable responses within the system (Gear et al., 2018c, 2019). Sometimes, it was used to understand the multidirectional interactions and patterns of nonlinear phenomena (Burge et al., 2014, 2016, 2019; Gear et al., 2018b, 2019; Katerndahl et al., 2019b, 2020; Makleff et al., 2020; Olive, 2017a, 2017b). For instance, using complexity science to gain a deeper understanding of the nonlinear dynamics of DVA and compare models of partner violence, such as the cycle of violence, family systems theory, and Duluth model, with mathematical models of complex dynamic patterns, such as periodic, chaotic, and random dynamics (Burge et al., 2014, 2016).

Complexity science could also be used to help understand how individual-level behavior can emerge at the community or societal level and was used to compare and explore possible outcomes and the consequences of various situations or interventions (Guidi et al., 2016; Hovmand & Ford, 2009a; Hovmand et al., 2009; Katerndahl et al., 2019a, 2020). It was used to explore the timing of community interventions, revealing that the sequence of implementation may be critical to the overall success of projects. For example, implementing a community intervention before introducing a mandatory arrest policy may yield better results (Hovmand & Ford, 2009a). At times, it was used to understand feedback mechanisms (Foot et al., 2015; Hovmand et al., 2009). Another function was to address barriers to collaboration and to help facilitate the use of diverse perspectives in the research (Deutsch et al., 2020; Hovmand et al., 2012). Finally, it was used to argue and advocate for a paradigm that has the potential to bring about transformational change if it can amalgamate multiple areas of lived experience and deep knowledge (Carne et al., 2019).

Hovmand and Ford (2009b) used “real options analysis” combined with “systems dynamic modeling,” as a complexity science method. Real options were an idea that came from corporate finance. It promotes a flexible strategy where investors who are not committed to accomplishing goals, can delay decisions when faced with uncertainty, and shift direction with changing circumstances. They explain that implementing community interventions can create localized uncertainty, as stakeholders may have conflicting agendas. A mandatory arrest policy may be resisted by those who view it with suspicion due to racial tensions or DVA advocates who fear it may lead to an increase in victim arrests. Systems dynamics modeling/real options can be used to explore the impact of introducing localized policies so that communities do not become expensive social experiments with potentially harmful consequences.

### Outcomes of Included Papers

Outcomes relate to the findings and recommendations of the studies. Outcomes varied, given the heterogeneous nature of the included studies.

Some papers found complexity science could capture new insights (Carne et al., 2019; Gear et al., 2018a). For example, using the research method “discourse analysis” (Gear et al., 2018b) conceptualized discourse as an emergent feature of CASs, created by the interactions between the systems agents, events, and the documents being analyzed. This highlights the continuous construction of knowledge and how it is understood, deployed, and adapted by different agents. Others found complexity science could identify and measure problems within the system of interest, such as competing agendas leading to system gaps and unintended consequences (Foot et al., 2015; Gear et al., 2018b) or that a lack of certainty and recognition of DVA challenged primary care responsiveness (Gear et al., 2019). Hovmand and Ford (2009a) also demonstrated that unintended consequences in community interventions were likely to emerge if complexity was not considered and only single outcomes were focused on. Complexity science could help promote healthier systems, such as sustainable responses in primary care (Gear et al., 2018c).

Makleff et al. (2020) found that complexity science was useful for understanding how community-level interventions, such as DVA programs run in schools, could disrupt the societal level. Hovmand et al. (2009) and Hovmand et al. (2012) demonstrated that societal-level policy could also negatively impact individuals, such as in the case of mandatory arrest policy for DVA, leading to more victims being unintentionally persecuted and a breakdown of the relationship between police and support workers.

Olive (2017a) focused on individual-level health consultations and found the social construction of wicked problems, such as DVA or acute patient stress reactions from the trauma of experiencing DVA (Olive, 2017b), could impact interactions and experiences during health care consultations. Papers also found that day-to-day DVA can be modeled (Burge et al., 2019; Katerndahl et al., 2019a, 2020) and that testing dynamic patterns in abusive relationships in real-time could relate to existing theoretical models, such as the cycle of violence, family systems theory and Duluth model (Burge et al., 2016). Katerndahl’s et al. (2019b) complexity science paper found that a pen-and-paper tool to access nonlinearity and unpredictability in DVA relationships was viable and reliable. Coordinated community responses to DVA have historically been challenging due to stakeholders having strong ideological and political reasons to protect their perspectives. It was found that complexity science methods, such as group model building, could also be a helpful asset in identifying and bridging diverse stakeholder tensions (Deutsch et al., 2020; Hovmand et al., 2012).

Many different recommendations emerged from the papers, aimed at clinical settings, researchers, policymakers, and society. Olive (2017a) suggested that the thresholds for the identification of levels of violence in clinical settings, such as emergency departments, were too high, and that all reports of DVA should be responded to with an intervention. It is important to understand the discordance of male and female versions of abusive events (Burge et al., 2019). Victims require privacy, safety, psychological first aid, a chronic care approach, and a fast response (Burge et al., 2014; Hovmand & Ford, 2009a; Olive, 2017b). They may also need different, couple-specific interventions for various patterns of abuse they have experienced (Burge et al., 2016; Katerndahl et al., 2020). Reliable tools are required to identify such patterns (Katerndahl et al., 2019b). Bystanders may need to modify their behavior to reduce DVA in society, and engaging male bystanders is particularly important (Guidi et al., 2016).

Complexity science could be useful and innovative to researchers exploring CASs and trying to understand the interdependencies of multiple issues (Gear et al., 2018a, 2018c; Hovmand & Ford, 2009b). Complexity science can be used in real-time or subsequently for analysis (Makleff et al., 2020). It can help address the underlying causes of complex problems and encourages us to become more comfortable with uncertainty at policy and practice levels (Gear et al., 2019). Complexity science holds great promise for policymakers who may recognize that challenging issues are related to societal dynamics (Hovmand et al., 2009). Lessons need to be shared internationally (Gear et al., 2018b), and long-term commitment and investment in the paradigm are necessary to realize its potential (Carne et al., 2019).

## Discussion

### Critical Findings

The findings demonstrated an increasing trend in the number of DVA complexity science articles, with most published by interdisciplinary teams in health or complexity science journals. Papers often incorporated multiple perspectives (e.g., feminist and psychological) and used complexity science as a “lens” to apply methods (e.g., systems dynamics, agent-based modeling, discourse analysis, or interviews) with complexity science strongly embedded into the paper. The journals that published complexity science DVA literature tended to be connected to the areas of health or complexity science. The included papers were also mostly cited in work published outside of DVA journals.

### The Global Disparity of Publications

The systems science papers included in the Tracy et al. (2023) review had mainly all originated from the United States. However, as this current review included “complexity theory” within the search terms, there were also a number of papers originating from New Zealand. Almost all the papers in both reviews came from higher-income countries, highlighting the disparity in the use of complexity science at a global level. The papers and their citations are mostly published in health and complexity science journals, which may indicate that audiences who are less familiar with DVA could be accessing them.

New Zealand has regularly adopted a complexity perspective in health research, policy, and governance (Eppel et al., 2011; Van der Heijden, 2020; Walton, 2016). Several published sources from New Zealand have been calling for a systems approach to address DVA, as the current approach appears to be failing victims of DVA. Without clarity about the interconnections across the system, they highlight that attempting to fix one part of a CAS in isolation could reveal or create unexpected problems downstream (Carne et al., 2019).

There have only been two papers published relating to DVA, using complexity science in a limited manner, originating from the United Kingdom Olive (2017a, 2017b). This may be due to the dominance of the feminist perspective at a theoretical and policy level, which tends to be grounded in the theory that DVA is predominantly caused by gender inequality and patriarchy (Bancroft, 2003; Dobash and Dobash, 1979).

### From Advocation to Application

Previous reviews have indicated an increasing use of complexity science within public health since 2005, with approximately 10 papers published in 2005 to over 80 publications in 2014 (Rusoja et al., 2018). Tracy et al. (2023) also noted a rapid increase in DVA/complexity science papers between 2013 and 2022, mirrored in our work.

However, a difference was noticed in how complexity science was used within the studies. Public health reviews indicated that most papers use complexity science as a conceptual framework or a position piece (Thompson et al., 2016). Carey et al. (2015) went on to state that there was a lack of engagement in applying complexity science on a pragmatic level. Almost half of the 122 papers in the Carey et al. (2015) review were position pieces that advocated how complexity science may be applied to public health or argued for its uptake. In contrast, in this review of the DVA literature, most papers used complexity science as a methodological approach.

This review’s findings highlighted some novel and interesting uses for complexity science to be utilized as a methodological approach. For example, the work by Gear et al. (2018b) may appeal to social scientists interested in applying a complexity lens to discourse analysis to understand how agents, documents, and events may interact with each other to create or change discourse, while work by Hovmand and Ford (2009b) or Katerndahl et al. (2019a) may be of interest to those interested in computer modeling. Group model building can help to provide transdisciplinary perspectives. This can be used to feed into systems dynamic modeling which may help to understand and simulate the impact on local interventions. The generic concepts of complexity science may help to pave a path for different disciplines to gravitate toward, understand, and appreciate one another’s work.

Complexity science is an alternative holistic perspective for approaching complex problems that can complement reductionist methods rather than replace them. The authors of the included papers often went to great lengths to help readers understand the paradigm and the importance of positioning and explaining research in this context (Gear et al., 2019; Hovmand & Ford, 2009a). Perhaps this is because complexity science is a less familiar paradigm, so an enhanced level of justification was deemed necessary, as many readers may be more familiar with a reductionist viewpoint.

### Diversity and Transdisciplinary Research

Complexity science places emphasis on listening to multiple perspectives (Deutsch et al., 2020; Hovmand et al., 2012). Diversity of views is important within CASs. Diversity supports novelty and innovation, and diverse, CASs tend to be more robust, allowing for multiple responses to external shocks and internal adaptations (Page, 2010). A lack of diversity could help create a *path-dependent* philosophy that can widen the gap between an authentic and delusory world (Gaub, 2019; Page, 2010). Once people have aligned themselves with strong beliefs, they seek ideas and people that support their values rather than factual evidence (Lord et al., 1979). This is known as “tribal epistemology,” which not only influences our thoughts about the world but also how we perceive and experience it (Dutton, 2020). Siloed working in academia may promote path dependencies in ideologies and practices.

Organizations supporting DVA victims are increasingly engaging with multiple tools and frameworks to enhance their support (Splitz, 2021), and it will be increasingly important to have academic paradigms that can reflect this transition and help create an alignment between the front-line work and the academic rhetoric. Transdisciplinary research is particularly important in domestic abuse research. Listening to the experiences and views of those who have experienced DVA themselves, particularly from under-represented backgrounds, to help close gaps, and keep research as authentic and as relevant as possible (Deutsch et al., 2020; Hovmand et al., 2012).

The included papers did not tend to have a strong DVA perspective stated. This could partly be due to the interdisciplinary nature of the papers. For example, Guidi et al. (2016) consisted of experts from across information engineering, education, psychology, physics, astronomy, and the study of complex dynamics. Complexity science demonstrated an ability to combine theoretical perspectives, and several papers used feminist and psychological ideologies within the articles (Burge et al., 2016; Guidi et al., 2016). Male victims were under-represented in the research, which aligns with the general trend that male victims are unrepresented in DVA research and service provision (Broberg, 2023). It may be easier to recruit female participants as service provision tends to be aimed at women and children.

The use of language is important when trying to encourage inclusivity and diversity in DVA. Terms, such as gender-based violence, which is often linked to the feminist perspective, or intimate partner violence may be suitable for some research; however, its use may encourage the exclusion of certain victims or perspectives. The papers from New Zealand seem to have framed DVA as a public health issue and have used terms, such as family violence, which may assist in DVA research becoming inclusive to a greater number of victims of DVA. The use of such language could support the combining of DVA perspectives, creating novel ideas and connections, and paving the way for all-encompassing principles and practice.

### Future Potential for Complexity Science in DVA Research

Complexity science could hold a great deal of potential for DVA research. Abusive and non-abusive networks often live side by side, merging at times (Shearing & Portal, 2021). Sometimes these networks are visible, while operating covertly to others. The form in which abuse emerges will depend on the interdependent relationships and the context of the situation. The interactions that can lead to patterns or emergent phenomena may vary depending on local circumstances (Waldrop, 1993). For example, different areas may have various levels of family support, cultural acceptance, or deterrents toward DVA, provoking diverse interactions and outcomes to emerge. Understanding networks and feedback in the systems, which can create or reinforce abuse, the interconnectedness of abuse, and the different forms abuse can emerge in, could help society intervene in ways that are currently not possible. Table 4 summarizes the potential implications of using complexity science for research, policy, and practice.

**Table 4.** Implications for Research, Policy, and Practice.

Implications for research	• CS supports a holistic perspective while capturing agent interactions that can lead to altered system outcomes. • CS could combine different perspectives, such as family systems theory and feminism. • CS encourages interdisciplinary teams to work collaboratively around an issue such as DVA.
Implications for policy	• CS could help to identify leverage points to assist in targeting scarce resources. • CS could help to predict the emergent phenomena and unexpected consequences of policy change.
Implications for practice	• CS may help to close the gap between academic research and front-line practice. • CS could support diverse (service user or stakeholder) voices to be heard in research.

Papers included in this review (Foot et al., 2015; Gear et al., 2019; Hovmand et al., 2009) help emphasize areas for potential change within the system. Still, individual, organizational, political, and societal collaboration and commitment are needed to implement the changes and achieve transformational change.

### Strengths and Limitations of the Study

This study builds on the work of previous public health and DVA reviews (Carey et al., 2015; Rusoja et al., 2018; Sturmberg et al., 2014; Thompson et al., 2016). It highlights not just the use of complexity science in DVA literature, but also emphasizes many pragmatic and varied uses of complexity science as a method. There is a wide range of search terms and language used in complexity science across many disciplines, which is constantly evolving, and as such, the use of our specific search terms may mean that some relevant articles were missed (e.g., around “Complexity Thinking”). Relevant information may be in the body of the full text only, meaning that they would not have been identified, as only headings and abstracts were screened, and so it is likely that some studies will have been missed. Relevant and worthwhile studies may have also been excluded if they were not written in English. Researchers are divided on their opinions on using “Google” as a search tool (Mahood et al., 2014). In this current review, Google was included as a search engine; therefore, this part of the study would be difficult to replicate. There was no input in the study from different stakeholder groups, such as people with lived experience of DVA. One author screened and extracted the data. This may impact on the reliability of the findings.

## Conclusion

This review has found that despite the modest number of published articles, complexity science can help us gain new understandings and perspectives. It also highlighted a broad spectrum of different ways that complexity science can be embedded into research, such as via theoretical and analytical lenses or as a position piece advocating how it may be utilized. It also demonstrated a variety of ways of utilizing complexity science as a research method.

Complexity science appeals to a broad range of disciplines and interests and emphasizes interdisciplinary teams and multi-stakeholder perspectives. Most included authors have a health-related background, but as an interdisciplinary paradigm and language, it can also appeal to other disciplines, such as engineers or computer scientists, encouraging diversity among researchers who may bring fresh insights to research. Policymakers are increasingly aware of the complex nature of problems; they are likely to be receptive to a holistic paradigm that can provide sustainable recommendations or cost-effective interventions, which may make a significant difference.

## Supplemental Material

sj-docx-1-tva-10.1177_15248380251316225 – Supplemental material for Complexity Science in Domestic Abuse Literature: A Systematic Scoping Review Supplemental material, sj-docx-1-tva-10.1177_15248380251316225 for Complexity Science in Domestic Abuse Literature: A Systematic Scoping Review by Sarah Blake and James Nobles in Trauma, Violence, & Abuse
