# Supplementary material for: Complexity Science in Domestic Abuse Literature: A Systematic Scoping Review
Source: Trauma Violence Abuse. 2025 Feb 24;27(2):448–60. doi: 10.1177/15248380251316225 (PMC12953660; doi:10.1177/15248380251316225)
Supplement: sj-docx-1-tva-10.1177_15248380251316225 – Supplemental material for Complexity Science in Domestic Abuse Literature: A Systematic Scoping Review [file sj-docx-1-tva-10.1177_15248380251316225.docx]

**Supplementary material for article: Complexity science in domestic abuse literature: A systematic scoping review**

**Supplementary material A: Search terms**

**Database search terms**

**Systems search terms**

“systems thinkin*” OR “Complexity theor*” OR “complex adaptive system*” OR “complexity science” OR “system* science” OR “whole system*” OR “system* approach*” OR “complex system*” OR “system* modelling” OR “system* dynamics” OR “Nonlinear dynamic*” OR “system* theor*” OR “system* biology” OR "agent-based modelling" OR "agent-based modeling" OR "agent-based model"

**Domestic abuse search terms**

“coercive control” OR “DVA” OR “DV” OR “domestic violence” OR “domestic abuse” OR “intimate partner abuse” OR “spous* abuse” OR “spous* violence” OR “battered women” OR “battered woman” OR “battered female*” OR “battered male*” OR “battered wife” OR “battered wives” OR “battered partner*” OR “battered man” OR “battered men” OR “wife abuse” OR “husband abuse” OR “abused wife” OR “abused husband” OR “wife violence” OR “husband violence” OR “wife beating” OR “husband beating” OR “intimate partner violence” OR “IPV” OR “intimate partner abuse” OR “partner abuse” OR “partner violence” OR “Conjugal abuse” OR “Conjugal violence” OR “marital abuse” OR “marital violence” OR “woman abuse” OR “abused woman” OR “women abuse” Or “abused women” OR “abused man” OR “abused men” OR “man abuse” OR “men abuse” OR “family abuse” OR “family violence” OR “elder abuse” OR “elder violence” OR “marital aggression” OR “relational aggression” OR “relationship aggression” OR “partner aggression” OR “interpersonal violence” OR “intimate terrorism”

Psycinfo

(("systems thinkin*" or "complexity theor*" or "complex adaptive system*" or "complexity science" or "system* science" or "whole system*" or "system* approach" or "complex system*" or "system* modelling" or "system* dynamics" or "Nonlinear dynamic*" or "system* theor*" or "system* biology" or "agent-based modelling" OR "agent-based modeling" OR "agent-based model") and ("coercive control" or "DVA" or "DV" or "domestic violence" or "domestic abuse" or "intimate partner abuse" or "Spous* violence" or "battered women" or "battered female" or "battered male" or "battered wife" or "battered wives" or "battered partner" or "battered man" or "battered men" or "wife abuse" or "husband abuse" or "abused wife" or "abused husband" or "wife violence" or "husband violence" or "wife beating" or "husband beating" or "intimate partner violence" or "IPV" or "intimate partner abuse" or "partner abuse" or "partner violence" or "conjugal abuse" or "conjugal violence" or "martial abuse" or "marital violence" or "women abuse" or "abused woman" or "women abuse" or "abused women" or "abused man" or "abused men" or "man abuse" or "man abuse" or "family abuse" or "family violence" or "elder abuse" or "elder violence" or "marital aggression" or "relational aggression" or "relationship aggression" or "partner aggression" or "interpersonal violence" or "intimate terrorism")).ab.

"systems thinkin*" or "complexity theor*" or "complex adaptive system*" or "complexity science" or "system* science" or "whole system*" or "system* approach" or "complex system*" or "system* modelling" or "system* dynamics" or "Nonlinear dynamic*" or "system* theor*" or "system* biology" or "agent-based modelling" OR "agent-based modeling" OR "agent-based model" and "coercive control" or "DVA" or "DV" or "domestic violence" or "domestic abuse" or "intimate partner abuse" or "Spous* violence" or "battered women" or "battered female" or "battered male" or "battered wife" or "battered wives" or "battered partner" or "battered man" or "battered men" or "wife abuse" or "husband abuse" or "abused wife" or "abused husband" or "wife violence" or "husband violence" or "wife beating" or "husband beating" or "intimate partner violence" or "IPV" or "intimate partner abuse" or "partner abuse" or "partner violence" or "conjugal abuse" or "conjugal violence" or "martial abuse" or "marital violence" or "women abuse" or "abused woman" or "women abuse" or "abused women" or "abused man" or "abused men" or "man abuse" or "man abuse" or "family abuse" or "family violence" or "elder abuse" or "elder violence" or "marital aggression" or "relational aggression" or "relationship aggression" or "partner aggression" or "interpersonal violence" or "intimate terrorism"

**Google/ Google scholar search terms**

complexity science, complexity theory, systems thinking, systems science, system dynamics modelling with “domestic violence” OR “domestic abuse” OR “Domestic violence and abuse” OR “intimate partner violence” OR “gender-based violence” OR “family violence” OR “partner abuse”

| Google Scholar Advanced search TOTAL n = 6 | | |
| --- | --- | --- |
| With all the words | With the exact phrase |  |
| Complexity science | “Domestic Violence” OR  “Domestic abuse” OR  “Domestic violence and abuse” OR “Intimate Partner Violence” OR  “Gender Based Violence” OR “Family Violence” OR “Partner abuse” | Burge 2016 (duplicate) |
| Complexity theory | “Domestic Violence” OR  “Domestic abuse” OR  “Domestic violence and abuse” OR “Intimate Partner Violence” OR  “Gender Based Violence” OR “Family Violence” OR “Partner abuse” | Gear 2018 (duplicate)  Gear 2019 (duplicate) |
| Systems thinking | “Domestic Violence” OR  “Domestic abuse” OR  “Domestic violence and abuse” OR “Intimate Partner Violence” OR  “Gender Based Violence” OR “Family Violence” OR “Partner abuse” | Caffery 2017 (duplicate)  Carne 2019 (include in full text review)  Befus 2019 (duplicate) |
| Systems science | “Domestic Violence” OR  “Domestic abuse” OR  “Domestic violence and abuse” OR “Intimate Partner Violence” OR  “Gender Based Violence” OR “Family Violence” OR “Partner abuse” | None |
| Systems dynamics modelling/ modelling | “Domestic Violence” OR  “Domestic abuse” OR  “Domestic violence and abuse” OR “Intimate Partner Violence” OR  “Gender Based Violence” OR “Family Violence” OR “Partner abuse” | None |
| **Total included: 1** | | |

**Google search terms and results**

| Google Advanced search TOTAL n = 25 | | |
| --- | --- | --- |
| With all the words | With the exact phrase |  |
| Complexity science | “Domestic Violence” OR  “Domestic abuse” OR  “Domestic violence and abuse” OR “Intimate Partner Violence” OR  “Gender Based Violence” OR “Family Violence” OR “Partner abuse” | Burge, 2016  Cabello, 2020  Caffrey, 2017  Carne, 2019  Deutsch, 2020  Drigo, 2010  Gear, 2019  Gear, 2018  Gear, 2018  Gear, 2018  Gear, 2019  Gear, 2021  Gram, 2021  Guidi, 2016  Hunt, 2020  Katerndahl, 2019  Katerndahl, 2021  Katerndahl, 2010  Katerndahl, 2020  Lewis, 2014  Madeira, 2021  Makleff, 2020  Miller, 2015  Olive, 2017  Foot, 2015 |
| Complexity theory | “Domestic Violence” OR  “Domestic abuse” OR  “Domestic violence and abuse” OR “Intimate Partner Violence” OR  “Gender Based Violence” OR “Family Violence” OR “Partner abuse” |  |
| Systems thinking | “Domestic Violence” OR  “Domestic abuse” OR  “Domestic violence and abuse” OR “Intimate Partner Violence” OR  “Gender Based Violence” OR “Family Violence” OR “Partner abuse” |  |
| Systems science | “Domestic Violence” OR  “Domestic abuse” OR  “Domestic violence and abuse” OR “Intimate Partner Violence” OR  “Gender Based Violence” OR “Family Violence” OR “Partner abuse” |  |
| Systems dynamics modelling/ modelling | “Domestic Violence” OR  “Domestic abuse” OR  “Domestic violence and abuse” OR “Intimate Partner Violence” OR  “Gender Based Violence” OR “Family Violence” OR “Partner abuse” |  |
| **Total included: 1** | | |

**Supplementary material B: Search results and reasons for exclusion**

| **Reason for exclusion from data base search** | **Number** |
| --- | --- |
| No abstract found | 15 |
| Not in English | 4 |
| Child abuse focus | 10 |
| Sexual abuse | 2 |
| Ecological model | 11 |
| Family systems theory | 36 |
| Not domestic abuse | 71 |
| Not SS/ CT | 91 |
| Other systems | 43 |
| Total | 283 |
| **Database search Included for full text review** | **33** |

**Supplementary material C: Full text screening and exclusion reasons**

|  | Author | year | Title | Outcome | HS |
| --- | --- | --- | --- | --- | --- |
| 1 | Befus, D. | 2019 | Systems Thinking Tools for Identification, Assessment, Intervention, and Evaluation of Traumatic Brain Injury (TBI) from Intimate Partner Violence (IPV): Canadian Indigenous Women as a Paradigmatic Case | The library was unable to source a suitable print or electronic version via inter-library loans |  |
| 2 | Burge, S  (Burge *et al.*, 2014) | 2014 | Safely examining complex dynamics of intimate partner violence | Yes in final included list | yes |
| 3 | Burge, S  (Burge *et al.*, 2019) | 2019 | The Dynamics of Partner Violence and Alcohol Use in Couples: Research Methods | Yes in final included list | yes |
| 4 | Burge, S  (Burge *et al.*, 2016) | 2016 | Using complexity science to examine three dynamic patterns of intimate partner violence | Yes in final included list | Yes |
| 5 | Katerndahl  (Katerndahl *et al.*, 2019a) | 2019 | Agent-Based Modeling of Day-to-Day Intimate Partner Violence | Yes | yes |
| 6 | Katerndahl  (Katerndahl *et al.*, 2020) | 2020 | Modeling women’s need for action in violent relationships | Yes | yes |
| 7 | Katerndahl, D  (Katerndahl *et al.*, 2019b) | 2019 | Psychometrics of the Violence Nonlinear Dynamics Scale | Yes in final included list  <https://www.psychiatrist.com/pcc/impulse/violence-aggression/psychometrics-of-the-violence-nonlinear-dynamics-scale/> | yes |
| 8 | Caffrey, L | 2017 | The Importance of Perceived Organisational Goals: A Systems Thinking Approach to Understanding Child Safeguarding in the Context of Domestic Abuse | Excluded - Family violence focus against children |  |
| 9 | Carlos, D. M. | 2017 | The care network of the families involved in violence against children and adolescents: the Primary Health Care perspective | Excluded - Family violence against children |  |
| 10 | Castellani, B. | 2018 | Exploring comorbid depression and physical health trajectories: A case-based computational modelling approach | Excluded - Focus on depression. DVA link but not main focus |  |
| 11 | Cirone, J. | 2020 | A System Dynamics Model of Violent Trauma and the Role of Violence Intervention Programs | Excluded - Focus on interpersonal violence not DVA. |  |
| 12 | Conway, Pat | 2013 | Strengthening systems to prevent intimate partner violence and sexual violence | Excluded - Contains elements of social network theory but SS/CT not focus. |  |
| 13 | Figueredo, Aurelio José | 2018 | Intimate partner violence, interpersonal aggression, and life history strategy | Excluded - other systems |  |
| 14 | Gear, Claire  (Gear, Eppel and Koziol-Mclain, 2018a) | 2018 | Advancing Complexity Theory as a Qualitative Research Methodology | Yes | Yes |
| 15 | Gear, Claire  (Gear, Eppel and Koziol-Mclain, 2018c) | 2018 | Utilising complexity theory to explore sustainable responses to intimate partner violence in health care | Yes | Yes |
| 16 | Gear, Claire  (Gear, Eppel and Koziol-Mclain, 2018b) | 2018 | Exploring the complex pathway of the primary health care response to intimate partner violence in New Zealand | Yes | Yes |
| 17 | Gear, C.  (Gear, Koziol-Mclain and Eppel, 2019) | 2019 | Exploring sustainable primary care responses to intimate partner violence in New Zealand: Qualitative use of complexity theory | Yes | Yes |
| 18 | Gear, Claire | 2016 | Developing a response to family violence in primary health care: the New Zealand experience | Excluded - Not SS/CT. Only used CAS as a recommendation for other future studies |  |
| 19 | Hovmand, P  (Hovmand *et al.*, 2012) | 2012 | Group Model-Building 'Scripts' as a Collaborative Planning Tool | Yes | Yes |
| 20 | Hovmand, P  (Hovmand and Ford, 2009) | 2009 | Sequence and timing of three community interventions to domestic violence. | Yes | yes |
| 21 | Hovmand,  P  (Hovmand *et al.*, 2009) | 2009 | Victims arrested for domestic violence: Unintended consequences of arrest policies. | Yes | Yes |
| 22 | Hovmand, P.  (Hovmand, Ford and Ieee, 2009) | 2009 | COMPUTER SIMULATION OF INNOVATION IMPLEMENTATION STRATEGIES | Yes | Yes |
| 23 | Jarvie, W | 2011 | Working with Complexity: Community Engagement and the Murdi Paaki COAG Trial | Excluded - Not dva focus and not much mention of it |  |
| 24 | Makleff, S  (Makleff *et al.*, 2020) | 2020 | Applying a complex adaptive systems approach to the evaluation of a school-based intervention for intimate partner violence prevention in Mexico | Yes | Yes |
| 25 | McIntyre, J | 2006 | Healing Pathways: Rescuing the Enlightenment from Itself: Implications for Addressing Democracy through Improved Collaboration and Knowledge Management | Excluded – Requested from inter library loans. not DVA focus |  |
| 26 | Olive, Philippa  (Olive, 2017b) | 2017 | Classificatory multiplicity: Intimate partner violence diagnosis in emergency department consultations. | Yes | Yes |
| 27 | Olive, P  (Olive, 2017c) | 2017 | First contact: Acute stress reactions and experiences of emergency department consultations following an incident of intimate partner violence. | Yes | Yes |
| 28 | Robertson, M | 2013 | Associations between the Life Course Development Model of Community Capacity Building and Abuse among Children and Adolescents in Washington State | Excluded - Focus on child abuse |  |
| 29 | Sexton, T | 1994 | SYSTEMIC THINKING IN A LINEAR WORLD - ISSUES IN THE APPLICATION OF INTERACTIONAL COUNSELING | Excluded – family systems therapy |  |
| 30 | Stewart | 2011 | Working with complexity: community engagement and the Murdi Paaki COAG trial 2002-2007 | Excluded - Duplicate of Jarvie article listed above |  |
| 31 | Foot J  Carswell S  Wood D  Nicholas G  (google)  (Foot *et al.*, 2015) | 2015 | measuring the effectiveness of 'whole of system'  response to prevent family violence | Yes | yes |
| 32 | Carne, S  David Rees  Nicola Paton  Janet Fanslow  (google scholar)  (Carne *et al.*, 2019) | 2019 | Using systems thinking to address intimate  partner violence and child abuse in New  Zealand | Yes | Yes |
| 33 | Deutsch, Lustfield, Jallali  (Deutsch, Lustfield and Jalali, 2020) | 2020 | Community-based system dynamics modelling of sensitive public health issues | Yes | Yes |
| 34 | Rigby, S. W.  Johnson, L. F. | 2017 | The relationship between intimate partner violence and HIV: A model-based evaluation | Excluded – HIV focus |  |
| 35 | Katerndahl, D.  Burge, S.  Ferrer, R.  Wood, R.  Montanez Villacampa, M. D. P. | 2020 | Effect of Incorporating Catastrophic Equations into an Agent-Based Model of Women's Action-Taking in Violent Relationships | Request submitted to library  Excluded – Not located |  |
| 36 | Guidi  (hand search)  (Guidi *et al.*, 2016) | 2018 | Stochastic Agent-based models of IPV | Yes |  |

**Supplementary material D: A summary of the disciplines identified by the authors of the papers**

| Discipline |  | Authors (n) |
| --- | --- | --- |
| Health  Total = 57 (73%) | Interdisciplinary trauma research | 4 |
|  | Violence research | 1 |
|  | Public health and policy | 1 |
|  | Family planning | 1 |
|  | Health services research | 2 |
|  | Health science centre | 6 |
|  | Veteran health care system | 1 |
|  | Social work | 5 |
|  | Behavioural sciences | 1 |
|  | Paediatrics | 1 |
| Engineering  Total = 4 (5%) | Information engineering | 1 |
|  | Civil engineering | 3 |
| Education  Total = 1 (1%) | Education and psychology | 1 |
| Physics  Total = 2 (3%) | Physics and astronomy | 1 |
|  | Nuclear physics | 1 |
| Environmental Science  Total = 3 (4%) | Environmental Science and Research | 1 |
| Complexity science  Total = 1 (1%) | Complex dynamics | 1 |
| Technology  Total = 1 (1%) | Technology assessment | 1 |
| Leadership  Total = 5 (6%) | Government | 4 |
|  | Management | 1 |
| Research  Total = 2 (3%) | Independent researcher | 2 |
| Business  Total = 2 (3%) | Independent businesses | 1 |

**Supplementary material E: Journals of the included papers**

Journal publications come from International Journal of Qualitative methods (Gear, Eppel and Koziol-Mclain, 2018), Health, Research, Policy and Systems (Gear, Eppel and Koziol-Mclain, 2018), Public Management Review (Gear, Eppel and Koziol-Mclain, 2018d), BMJ Open (Gear, Koziol-Mclain and Eppel, 2019), Health Policy and Planning (Makleff et al., 2020), Journal of Clinical Nursing (Olive, 2017, Olice 2017) , Family Systems and Health (Burge et al., 2014, Burge 2016), Nonlinear Dynamics, Psychology, and Life sciences (Katerndahl et al., 2019), Journal of Interpersonal Violence (Katerndahl et al., 2020), Violence and Victims (Burge et al., 2019), American Journal of Community Psychology (Hovmand and Ford, 2009), Systems Dynamics Review (Hovmand et al., 2009), Systems Research and Behavioural Science (Hovmand et al., 2012). The remaining six papers were published via other sources and independent websites (Katerndahl et al., 2019, Guidi et al., 2016, Hovmand and Ford, Deutsch, Lustfield and Jalali, 2020, Foot et al., 2015, Carne et al., 2019).
